# Pathology of Fevers

**Published:** 1855-09

**Authors:** N. S. Davis

**Affiliations:** Prof. of Principles and Practice of Medicine, and Clinical Medicine; in Rush Med. College; Member of American Med. Association, &c., &c.


ARTICLE VIII.—*The Pathology of Fevers; or Observations Critical and Experimental, in reference to the nature, varieties, causes, and treatment of Fevers.* By N. S. DAVIS, M. D., Prof. of Principles and Practice of Medicine, and Clinical Medicine; in Rush Med. College; Member of American Med. Association, &c., &c.

CAUSES OF FEVER, (*Continued from July Number.*)

THE facts adduced in regard to the amount and kind of matter eliminated from the skin and lungs, are abundantly sufficient to demonstrate the great importance of these matters as direct exciting causes of disease. As such, they are operative, not merely in the narrow alleys, and crowded streets of our large cities, but often also in the most thinly populated rural districts. We are so accustomed to associate together the ideas of *pure air* and a *country residence*, that we are liable to overlook the most prominent ininstances of impurity, provided they be located in the country districts. On a careful examination, however, it will be found that throughout most of the rural districts of our country, the sleeping apartments are very small, and often provided with very inadequate means for ventilation.

The farm houses generally are low between floors, and a large proportion of bed-rooms only *nine* by *twelve* feet or less. The chambers, especially, in which most of the hired help and the younger members of the family sleep, are often only *half a story* between the floor and the roof, with only small windows at the ends.

Let any one compare the actual capacity of such rooms with the amount of gaseous materials, impregnated with animal matter, which escape from the inmates during the hours of their occupancy, and after making all proper allowance for the degree of ventilation that actually exists, he will be forced to the conclusion that a large proportion of those who reside in the country, as well as those in the cities, habitually inhale during the night an atmosphere too impure to be consistent with the maintenance of good health. Persons thus situated during the night, generally rise in the morning with a feeling of languor and heaviness, that disappears only, after from one to two hours exposure in the open air; and they generally have much less relish for breakfast than for dinner or supper.—The deleterious effects here described may not often be sufficient to induce a direct attack of fever or any other acute disease But that they debilitate or depress the vital properties and elementary functions of the system to such an exent as to constitute a strong *predisposition* to such diseases, I have no doubt. About four years since, during the winter season, a series of religious meetings were held for many successive days and evenings, in a small, ill-ventilated school-house, about sixteen miles from this city. Much interest was felt by the families in the neighborhood and the house was crowded most of the time during the meetings. Before this season of religious revival ended, *pneumonia* accom panied by well-marked *typhoid* symptoms began to prevail among the people, and several cases terminated fatally. By direct inquiries at the time, it was ascertained that most of those attacked had been among the most constant attendants in the crowded meetings referred to; and among the fatal cases were some of the most promising young men of the neighborhood.

In referring to this, as an illustration of the effects of air renered impure, by accumulated exhalations from the crowded assemblies, let no one infer that I object either to religious meetings, or even *revivals.*—They should be conducted, however, with proper regard to the laws of health. Late in the autumn of 1851, I was called into the country twelve miles, to visit the son of Mr. C., who I found dangerously ill with typhoid fever, complicated with disease of the lungs. Mr. C., though the owner of a large farm, situated on a wide rolling prairie, still lived in the old *log-house*, which had given him shelter when he first settled in the country. The body of the house furnished but one room, which served the purposes of kitchen, parlor, and dining-room for the whole family, and in one corner of which was placed a bed surrounded by curtains. In this the parents usually slept, but it was now occupied by the sick son. The rest of the family, numbering eight or nine persons, slept in the low chamber, ventilated only by a small window at each end.

Nearly all the members of the family were complaining of those feelings of indisposition, and that loss of appetite in the morning, which was clearly traceable to the impurity of the air in which they slept. And I am satisfied that nothing but the modifying influences of habit and the free exposure to the air during the day, prevented them from all becoming sick with typhoid disease. That the vital properties of the human system are capable of becoming adapted, by repeated exposure, to the impressions of an atmosphere more or less impure, so far as to maintain the several functions in a condition compatible with fair health, I have no doubt. But that the standard of health in such cases is less perfect, and the vital properties less capable of resisting the influence of ordinary exciting causes of disease, is equally certain. Persons thus situated are consequently, in a strict sense, constantly more or less *predisposed* to disease: and more readily succumb under the influence of any of the more active morbific agents. This is illustrated during the prevalence of almost every epidemic disease. The efficient cause or causes of such diseases are generally conceded to act on whole communities at the same time. But the investigations of sanitary commissions and Boards of Health, as well as the experience of observing physicians every where, have clearly shown that those who live in such places as possess an atmosphere most confined, impure, and damp, are not only the first to suffer attacks, but in them the disease is much more likely to prove fatal. And yet even in such localities, and during the prevalence of typhoid fever, typhus, dysentery, and cholera, we may see the influence of habit or continued exposure; those who have been long residents in the same place being less liable to attacks than the new comers. It was long since shown by Louis, Chomel, and other eminent writers that a large proportion of the cases of typhoid fever occurring in Paris and other European cities, took place in those persons who had recently changed their residence from the rural districts to the city.

The same fact has been noticed in the cities of our own country — During the last four years, I have kept a record of two hundred and fifty cases of typhoid fever, which have occurred under my own observation in this city. Of these, two hundred and five, or little more than four-fifths occurred in persons who had been less than one year residents in this city. Many of them were young men from the rural districts of the middle and eastern States, accustomed to pure air and free out-of-doors exercise.

On coming to the city, they become occupied chiefly in shops, stores, and offices, take much less out-door exercise than formerly, sit up later in the evening, eat less regularly, and often patronize the *saloons* after the regular duties of the day are over, while the whole atmosphere of the city is less pure, and consequently less invigorating than that to which they had been previously accustomed. The atmosphere inhaled into the lungs constitutes at once the great excitor or stimulus, which, acting through the blood on the elementary susceptibility of all the the tissues of the body, maintains their natural and healthy actions or functions, while on the other hand, it furnishes a medium in which is carried off a large amount of effete matter in the form of carbonic acid, water, and animalized substances. Hence every change in the atmosphere is liable to affect, not merely the properties or functions of a single organ, but of all the organs and tissues of the body.

If by increasing the density or other means, the quantity of oxygen in a given number of cubic-inches of inhaled air is increased, it is very probable that a greater amount would enter the blood, causing a more active excitation of all the tissues, a greater rapidity of organic or molecular change, and an exaltation of all the properties and functions of the system. Every individual feels something of this, whenever exposed to a dry, cool, and pure atmosphere. On the contrary, by diminishing the quantity of oxygen in a given quantity of inhaled air, exactly the reverse effect is induced. A less quantity of oxygen is absorbed ; excitation is difficient, and consequently the molecular changes take place less rapidly. An individual in such a condition feels; mentally, listless and dull, and throughout the muscular system a sense of lassitude or indisposition to exertion is predominant. These effects may be experienced at any time by inhaling an atmosphere made very *rare* either by its high temperature or its altitude. If we examine the individual under such circumstances more carefully, we shall soon find that the diminished inhalation of oxygen has not merely diminished the tone and activity of the muscular and nervous tissues, but the same deficiency of the natural stimulus or excitor in the blood has diminished the affinity of the blood for the cells of the various secreting structures. Hence at the same time that the patient becomes conscious of mental inactivity and muscular lassitude all the secretions are diminished, causing the skin to be more dry, the urine to be diminished in quantity, and generally a deeper color, the fœces to indicate deficient coloring matter of bile, while the loss of appetite or imperfect digestion of food generally shows deficient secretion of the gastric fluid.—The same altered affinity between the blood and the tissues, also impairs those molecular movements by which effete or waste particles are removed and new ones appropriated thoughout all the tissues of the body The former consequently accumulate more or less rapidly, until their presence excites a responsive action in some one of the eliminating organs through which they escape, mingled with the cutaneous, renal, or intestinal secretions; or failing in this, they act as irritants upon tne elementary properties of the the tissues, exalting the susceptibility and all the ordinary phenomena of febrile action.

It is thus, that a simple change in the constituents of the air we breathe, by lessening even in a slight degree the usual stimulus to the elementary properties of organized matter, may lead to the simultaneous disturbance of every function in the system. This disturbance may be so slight as to cause only temporary feeling of indisposition or absence of vigorous health, or it may be so great as to speedily develope a grave general fever. It is precisely this general disturbance, or more properly, general depression of all the functions arising from a diminution of the oxygen inhaled with a given number of cubic inches of atmospheric air that is felt in some degree by almost all persons when they remove from colder climates to warmer ones, and from rural districts to densely populated cities. The process of "acclimation," as it is called, is simply the period necessary for the elementary properties of the tissues, and consequently all the functions dependent on these properties, to become adapted to the impressions of an atmosphere different in its temperature, density or other conditions, from that to which the individual had been previously accustomed. If the difference in the atmospheric conditions is only slight, as is often the case when the individual only removes from a country district into a city in the same latitude, the great majority will suffer so little that they require no attention. But ever here, if in addition to the influence of a moderate change in the conditions of the atmosphere, the individual after arriving in the city, enters upon a business which confines him more than usual within doors, and causes him to be more irregular in his habits of eating, drinking, and rest, these will constitute so many additional influences calculated to increase the depression of the vital properties and more certainly cause the functional disturbance to be sufficient to constitute disease. From much careful observation on this subject, I am fully satisfied that the chief causes which induce in so large a proportion of those young persons who resort to our cities either for study or business, attacks of typhoid or continued fever during the first year of their residence, are those just named. I am aware that Dr. Watson of London, and many others, strenuously contend that all our attacks of typhoid or common continued fever depend essentially upon some specific "fever poison;" which they are compelled to regard as existing almost everywhere, and at all seasons of the year, especially in cities, but which is capable of isolation or tangibility nowhere. I frankly confess that I can see neither good sense nor sound philosophy in calling to our aid a purely hypothetical agent, and making it play an important, almost protæan, part in the causation of disease when all the phenomena produced in connection with the sick can be fully explained by changes in the known elements and conditions around us. I do not deny the propriety, or even necessity of sometimes resorting to the supposition that agencies exist, not yet rendered tnagible to us, and therefore hypothetical.

It may be necessary to infer the existence of a cause, merely from its *effects;* but I protest against doing this in all instances connected with medical science, where *effects* can be fairly accounted for by the influence of known agencies. That patients laboring under continued fever, either of the typhus or typhoid varieties, may sometimes evolve with the eliminations from their bodies, an animal poison, capable of generating the same disease in others may be quite true. But the supposition that such poison constantly exists in the atmosphere of either city or country, and thereby constitutes the efficient cause of these diseases in all cases, is neither consonant with facts nor sound principles of induction.

On the contrary, a vast variety of facts might be accumulated, which show the direct connection of appreciable atmospheric conditions with the production of febrile diseases generally.

For instance, the symptoms already pointed out as the effects of habitual exposure to an atmosphere containing less oxygen in a given quantity of air than that in which the individual had previously lived, will be readily recognized as identical in kind, and and often in degree also, with those which are described by all authors as constituting the first, or forming stage of ordinary typhoid fever. It is not probable that this simple change in the constitutents of the atmosphere, is alone generally sufficient to develope febrile disease, but it so far modifies the vital properties and lessens the activity of the primary functions of the system, as to constitute a decided *predisposition*, I have no doubt.

And while this predisposition, or altered state of the properties and functions exists, the influence of the same collateral, or exciting causes which prove nearly harmless to others, will in them determine the development of active disease. For instance, an old resident of the city, with vital properties fully adapted to the impressions of its atmosphere may frequently be up late at night, eat irregular, confine himself to his counting-room, or frequent crowded public rooms, with only slight inconvenience or temporary debility; while the same habits indulged in by one just removed from the more invigorating atmosphere of a rural district, would almost certainly produce decided feelings of indisposition and in a large proportion of cases, if persisted in, would develope a well marked attack of continued fever before the end of the first eight months. On the the other hand, if all such collateral disturbing influences are carefully avoided, probably in nineteen cases out of twenty, healthy individuals may change from the country to the city, or from a colder to a warmer climate, with no other inconvenience than a moderate degree of debility or feeling of indisposition which disappears in a few weeks; and which amidst the novelty and excitement of the change are often not noticed at all.

Aside from the influence exerted by changes in the temperature and density of the atmosphere, there are two other agents equally potent for good or evil, and equally capable of modifying the elementary properties and functions of the system. These agents are atmospheric electricity and moisture.

Experiment has long since shown that electricity possesses an excitor influence over nerve matter, so direct and powerful that many physiologists have regarded it as identical with nerve force. There is reason to believe, however, that its action is not limited entirely to the nerve tissue ; but is exerted also on the elementary properties of all the tissues. Its influence in certain cases of retarded or suppressed menstruation, as well as its effects on capillary circulation generally, show it to possess an action not easily explained by any supposed modification of the nervous system or its influence. How much of the invigorating influence imparted by a cool and dry atmosphere, is owing to the electricity it contains, it is not possible in the present state of our knowledge to determine. . There is still an open and inviting field for investigation. We want some inventive genius to furnish us with the instruments capable of indicating readily the exact electrical condition of the atmosphere at any and all times ; and then we need a patient and continued series of recorded observations in regard to the prevalence and specific character of diseases, in comparison with the variations in the atmospheric electricity. Until within a few years the attention of physiologists has been confined almost wholly to the temperature of the atmosphere, and its admixture with vegetable or animal matters, the products of decomposition.

It is only within a very few years that reliable records of the variations in the quantity of aqueous vapor suspended in the atmosphere have been kept and examined in their etiological bearings. And we have a still smaller amount of reliable knowledge in regard the electrical conditions. Even the few facts we have on record in relation to this have been observed during some special prevalence of disease, and are unconnected with any similar records during periods of good public health, so as to admit of a just comparison.

